# TRPV4 Channel Inhibits TGF-β1-Induced Proliferation of Hepatic Stellate Cells

**DOI:** 10.1371/journal.pone.0101179

**Published:** 2014-07-11

**Authors:** Yang Song, Lei Zhan, Mingzhe Yu, Cheng Huang, Xiaoming Meng, Taotao Ma, Lei Zhang, Jun Li

**Affiliations:** 1 School of Pharmacy, Anhui Medical University, Hefei, China; 2 Institute for Liver Diseases of Anhui Medical University (AMU), Hefei, China; 3 The First Affiliated Hospital of Anhui Medical University, Hefei, China; National Institutes of Health, United States of America

## Abstract

TRPV4, one of the TRP channels, is implicated in diverse physiological and pathological processes including cell proliferation. However, the role of TRPV4 in liver fibrosis is largely unknown. Here, we characterized the role of TRPV4 in regulating HSC-T6 cell proliferation. TRPV4 mRNA and protein were measured by RT-PCR and Western blot in patients and rat model of liver fibrosis in vivo and TGF-β1-activated HSC-T6 cells in vitro. Both mRNA and protein of TRPV4 were dramatically increased in liver fibrotic tissues of both patients and CCl_4_-treated rats. Stimulation of HSC-T6 cells with TGF-β1 resulted in increase of TRPV4 mRNA and protein. However, TGF-β1-induced HSC-T6 cell proliferation was inhibited by Ruthenium Red (Ru) or synthetic siRNA targeting TRPV4, and this was accompanied by downregulation of myofibroblast markers including α-SMA and Col1α1. Moreover, our study revealed that miR-203 was downregulated in liver fibrotic tissues and TGF-β1-treated HSC-T6 cell. Bioinformatics analyses predict that TRPV4 is the potential target of miR-203. In addition, overexpression of miR-203 in TGF-β1-induced HSC significantly reduced TRPV4 expression, indicating TRPV4, which was regulated by miR-203, may function as a novel regulator to modulate TGF-β1-induced HSC-T6 proliferation.

## Introduction

Liver fibrosis is a medical problem worldwide with high morbidity and mortality, and endangers human health seriously [1]. Hepatic stellate cells (HSC) play an essential role in the development of liver fibrosis. For example, following fibrotic injury, HSC undergo transdifferentiation from quiescent vitamin-A-storing cells to an activated myofibroblastic phenotype identified by upregulation of α-smooth muscle actin (α-SMA) and type I collagen, thereby contributing to the progression of liver fibrosis [2], [3]. However, the molecular mechanisms responsible for the proliferation and activation of HSC are still unclear. In the case of HSC, this proliferation and activation responses can be prevented by treatment with chemical inhibitors of ion channels [4], [5]. But so far, the detailed mechanisms, by which ion channels regulate liver fibrosis, are complex and have not been fully elucidated.

Transient receptor potential vanilloid 4 (TRPV4) was firstly identified as a channel activated by hypotonicity-induced cell swelling, however, TRPV4 is also sensitive to a wide variety of physical and chemical stimuli [6]. Importantly, it is able to integrate different stimuli and confer many distinct cellular functions in various cell types throughout the body [7], [8], [9], [10]. Here, we detected increased TRPV4 mRNA and protein level in the liver of rats subjected to liver injury. The blockade of TRPV4 with Ruthenium Red (Ru) or TRPV4-siRNA inhibited the proliferation of activated HSC-T6 cells and decreased α-SMA and Col1α1 expressions. Moreover, we explored the potential function of miR-203 in the regulation of TRPV4 in vitro. Our results suggested a pathological role of TRPV4 in the activation and proliferation of HSC, indicating that TRPV4 may be a potential therapeutic target in the treatment of liver fibrosis.

## Materials and Methods

### 2.1 Materials and reagents

Non-tumorous portions of the liver were obtained from patients undergoing partial liver resection at the Department of Surgery, The First Hospital of Anhui Medical University. The degree of fibrosis was classified as normal liver and mild to moderate fibrosis according to the Liver Cancer Study Group of Japan. Written informed consent was obtained from all patients. The study was approved by the Ethical Committee of Anhui Medical University and followed the ethical guidelines of the Declaration of Helsinki.

CCl_4_ was obtained from Shantou Xilong Chemistry Plant (Shantou, China). Dimethylsulfoxide (DMSO) were purchased from Sigma Inc. (St. Louis, MO, USA). Mouse monoclonal antibodies against α-SMA and β-actin were obtained from Boster (Wuhan, China). TRPV4 antibodies were purchased from Abcam (Cambridge, UK). TGF-β1 was purchased from Peprotech (New Jersey, USA). miR-203, TRPV4, α-SMA and Collagen Ι primers were produced from Shanghai Sangon Biological and Technological Company (Shanghai, China). Ruthenium Red was purchased from Sigma (Deisenhofen, Germany). DNA extraction kit was acquired from Axygen. Streptavidin peroxidase (SP) immunohistochemical kit was acquired from Zhongshan Biotechnology Corporation (Beijing, China). Secondary antibodies for goat anti-rabbit immunoglobulin (IgG) horse radish peroxidase (HRP), and goat anti-mouse IgG HRP were purchased from Santa Cruz Biotechnology (Santa Cruz, California, USA).

### 2.2 CCl_4_ liver injury model

Liver fibrosis was generated by a 12-week treatment of adult male Sprague-Dawley (200–220 g) rats with CCl_4_ (CCl_4_/olive oil, 1∶1 (vol/vol) per kg body weight by intraperitoneal injection twice weekly) as previously described [11]. Vehicle control animals were treated intraperitoneally with 1 ml/olive oil/kg body weight at the same time intervals. 24 h after the final CCl_4_ injection, rats were sacrificed and liver tissues were harvested for the further analysis. Animals were provided by the Experimental Animal Center of Anhui Medical University. The animal experimental protocol was approved by the University Animal Care and Use Committee of Anhui Medical University.

### 2.3 Cell culture and cell treatment with TGF-β1

HSC-T6 cell line was obtained from Shanghai FuMeng Gene Bio-technology Co., LTD. (Shanghai, China). HSC-T6 cells were cultured in Dulbecco's modified Eagle's medium (DMEM, USA) supplemented with 10% fetal bovine serum, 100 U/ml penicillin and 100 mg/ml streptomycin in a humidified incubator at 37°C with 5% CO2. These cells were propagated for 48 h and serum-starved with 0.5% FBS for 24 h before adding 10 ng/ml Platelet-derived growth factor (PDGF-BB, peprotech, USA).

### 2.4 Cell transient transfection of miR-203 mimics and siTRPV4

HSC-T6 cells were cultured in serum-free DMEM for 12 h and then subjected to transfection with miR-203 mimics, (NS)-miRNA (GenaPharma, China) and Si-TRPV4, Si-control using Lipofectamine 2000 (Invitrogen, USA)according to the manufacturer's instruction. The culture medium was changed 6 h after transfection, and TGF-β1 (Peprotech, USA) was added at a concentration of 10 ng/ml. The sequences of oligonucleotides used are as follows:

miR-203 mimics: 5′-GUGAAAUGUUUAGGACCACUAG-3′

5′-AGUGGUCCUAAACAUUUCACUU-3

NS-miRNA: 5′-UUCUCCGAACGUGUCACGUTT-3′

5′-ACGUGACACGUUCGGAGAATT-3′

Si-TRPV4: 5′- CCGUGUCCUUCUACAUCAATT -3′

5′- UUGAUGUAGAAGGACACGGTT -3′

si-control: 5'-UUCUCCGAACGUGUCACGUTT-3'

5'-ACGUGACACGUUCGGAGAATT-3'

### 2.5 Ruthenium Red (Ru) treatment

Ru was dissolved in Dulbecco's modified Eagle's medium (DMEM, Gibco, USA) with 10% fetal bovine serum and used at a concentration of 1 uM. HSC-T6 cells were seeded overnight in culture dishes and treated with TGF-β1 plus Ru.

### 2.6 Semi-quantitativereversetranscription-polymerasechainreaction(RT-PCR)

Total RNA was extracted from rat liver tissues and HSC-T6 cells using TRIzol reagents (Invitrogen). The first-strand cDNA was synthesized from total RNA using Thermoscript RT-PCR System (Takara) according to the manufacturer's instructions. RT-PCR was carried out under standard protocol using the following primers: β-actin (forward: 5'-TGAGCTGCGTGTGGCCCCTGAG-3'; reverse: 5'-GGGGCATCGGAA CCGCTCATTG-3'), TRPV4(forward: 5'-CGCCTCCGCAGGGATCGCTGGTC-3'; reverse: 5'-TGAGCTGGCTTAGGTGACTCCATGGGAGTG-3'), α-SMA (forward: 5'-TGGCCACTGCTGCTTCCTCTTCTT-3'; reverse: 5'-GGGGCCAGCTTCGTCAT ACTCCT-3'), Col1a1: (forward: 5'-TACAGCACGCTTGTGGATG-3'; reverse: 5'-TT GAGTTTGGGTTGTTGGTC-3'). PCR was performed at 94°C for 5 min, followed by 35 or 38 cycles of amplification at 94°C for 36 s, 52 or 60°C for 36 s and 72°C for 1 min by using ABI9700. The band intensities were measured by a densitometer and the results were normalized with β-actin. The results were repeated at least three times independently from three different pools of templates, while each pool of template was extracted from at least three ventricles.

### 2.7 Quantitative real-time PCR

Total RNA was extracted from the cultured cells using a RISO RNA Isolation Reagent (Biomics, USA). Expression of miR-203 was measured using an EzOmics miRNA qPCR Detection Primer Set (Biomics, USA) and EzOmics One-Step qPCR Kit (Biomics, USA) in PikoReal 96 real-time PCR system (Thermo Fisher Scientific, Finland).The fold-change for miR-203 relative to U6 was calculated using the 2- ΔΔCt method, where ΔΔCt  =  ΔCt BEL/5-FU-ΔCt BEL-7402 and ΔCt  =  Ct miR-203-Ct U6. PCR was performed in triplicate. Total RNA was isolated from the cultured cells using the TRIzol reagents (Invitrogen, USA), and the first strand cDNA was synthesized using a ThermoScript RT-PCR synthesis kit (Fermentas, USA) according to the manufacturer's instructions. Quantitative real-time PCR analyses for mRNA of TRPV4, α-SMA, β-actin were performed by using the QuantiFast SYBR Green RT-PCR kits (QIAGEN, Germany). The mRNA level of β-actin was measured as an internal control.

### 2.8 RNA interference (RNAi) analysis

RNAi experiments in HSC-T6 cells were performed by forward transfection in day 2 cultured HSC-T6 (2×10^5^ cells per 200 mm^2^ dish) using Lipofectamine RNAiMax (Invitrogen) according to the manufacturer's protocol. For TRPV4 immunoblotting, HSC-T6 cells were cultured in serum-free DMEM for 12 h and then subjected to reverse transfection with RNAiMax in Opti-MEM. Small interfering RNA (siRNA) oligonucleotides against TRPV4 genes or scrambled sequences were synthesized by the Shanghai GenePharma Corporation. The following siRNA sequences were used: si-TRPV4 (rat), 5'-CCCAGAGUAUGCACCAAUATT-3' (sense) and 5'-UAUUGGUGCAUACUCUGGGTT-3' (anti-sense); si-control with scrambled sequence (negative control siRNA having no perfect matches to known rat genes), 5'-UUCUCCGAACGUGUCACGUTT-3' (sense) and 5'-ACGUGACACGUUCGGAGAATT-3' (antisense). Transfection was allowed to proceed for various times and cells were processed for different assays. The siRNA transfection efficiency of Lipofectamine RNAiMax in cells was determined by the BLOCK-iT Alexa Fluor Red Fluorescent Oligo protocol (Invitrogen).

### 2.9 MTT assay

Cellular proliferation was measured using MTT assay. 5×10^3^ cells were seeded in 96-well plates and cultured with siRNA-TRPV4 at 37°C in a humid chamber with 5% CO2 for 2 days. 5 mg/ml MTT was then added to each well and incubated with cells at 37°C for 4 h. After removal of supernatant, 150 ul of DMSO were added to each well. The optical density (OD) was measured at 490 nm. The percentage of viability was calculated according to the following formula: viability % = T/C×100%, where T and C refer to the absorbance of transfection group and cell control, respectively.

### 2.10 Western blotting

Rat liver tissues and cells were lysed with lysis buffer. Whole-cell extracts were prepared, and protein concentration of samples was determined using a BCA protein assay kit (Boster, China). Whole-cell extracts (30 or 50 mg) were then fractionated by electrophoresis through an 10% sodium dodecyl sulfate-polyacrylamide gel electrophoresis (SDS-PAGE). Gels were run at a 120 V for 2 h before being transferred onto a PVDF membrane (Millipore Corp, Billerica, MA, USA). After blockade of nonspecific protein binding, nitrocellulose blots were incubated for 1 h with primary antibodies diluted in TBS/Tween20 (0.075%) containing 3% Marvel. Anti-TRPV4 (Cell Signaling, Beverly, MA, USA) was diluted 1∶400, and rabbit monoclonal anti-α-SMA (Boster) was diluted 1∶200. Rabbit monoclonal antibodies directed against TRPV4 (Abcam, Cambridge, UK) or β-actin (Santa Cruz Biotechnology, CA, USA) were used at 1∶400. Following incubation with primary antibodies, blots were washed four times in TBS/Tween-20 before incubation for 1 h in goat anti-rabbit horseradish peroxidase conjugate antibody at 1∶10000 dilution in TBS/Tween-20 containing 5% skim milk. After extensive washing in TBS/Tween-20, the blots were processed with distilled water for detection of antigen using the enhanced chemiluminescence system. Proteins were visualized with the ECL-chemiluminescent kit (ECL-plus, Thermo Scientific).

### 2.11 Immunohistochemistry

Liver tissues were fixed in 10% neutral buffered formalin solution, embedded in paraffin, and stained for routine histology. The sections were dewaxed in xylene and dehydrated in alcohol, antigen retrieval was achieved by microwaving in citric saline for 15 min. Thin sections were deparaffinized and treated with 0.3% hydrogen peroxide for 15 min to block endogenous peroxidase activity. The sections were further blocked by 2% bovine serum albumin followed by incubation with primary antibody against TRPV4 (1∶400) for 16 h at 4°C. After rinsing, the sections were incubated with biotinylated secondary antibody for 60 min at room temperature. TRPV4 expression was visualized by 3,3'-diaminobenzidine tetrahydrochloride (DAB) staining. Slides were counterstained with hematoxylin before dehydration and mounting. TRPV4 positive areas within the fibrotic region were then observed. Quantitative analysis was calculated from five fields for each liver slice.

### 2.12 Luciferase reporter assays

Luciferase reporter assays were carried out in HSC. Briefly, HSC-T6 cells (5×104cells/well) were cultured into 24-well plates and co-transfected with 200 ng DNA of TRPV4-UTR wt plasmid in the presence of 60 nmol of miR-203 mimics and NS-miR-203 (Gene Pharma, Shanghai, China), using 2.5 uL lipofectamine 2000 and 100 uL Opti-MEM (Invitrogen, USA). The cells were harvested and lysed 48 h after transfection and the luciferase activities were measured consecutively by the Dual-Luciferase Reporter 1000 Assay system (Promega, USA). Renilla luciferase activity was normalized to Firefly luciferase activity. All experiments were performed independently in triplicate.

### 2.13 Double Immunofluorescent Staining

For double immunofluorescent staining, sections were firstly blocked with 10% normal serum blocking solution in order to avoid unspecific staining. Then, the sections were incubated with rabbit polyclonal primary antibodies for TRPV4 (1∶50; Abcam) and mouse monoclonal primary antibodies for α-SMA (1∶100; Santa Cruz). Sections were incubated with both primary antibodies over-night at 4°C, followed by a mixture of anti-mouse FITC (1∶200) and anti-rabbit TRITC (1∶200) conjugated secondary antibodies for 2 h at room temperature. Then the stained sections were examined with a Leica sp5 laser confocal microscope (Germany).

### 2.14 Statistical analysis

Data are represented as mean ± SE. Statistical analysis was performed using ANOVA followed by Student's t-test. For changes in mRNA or protein levels, ratios of mRNA (relative expression) and protein (densitometric values) to respective house-keeping controls were compared. Significance was defined as *p*<0.05.

## Results

### 3.1 Upregulation of TRPV4 mRNA and protein in the liver tissues from liver fibrosis patients and CCl_4_-treated rats

**Figure 1:**
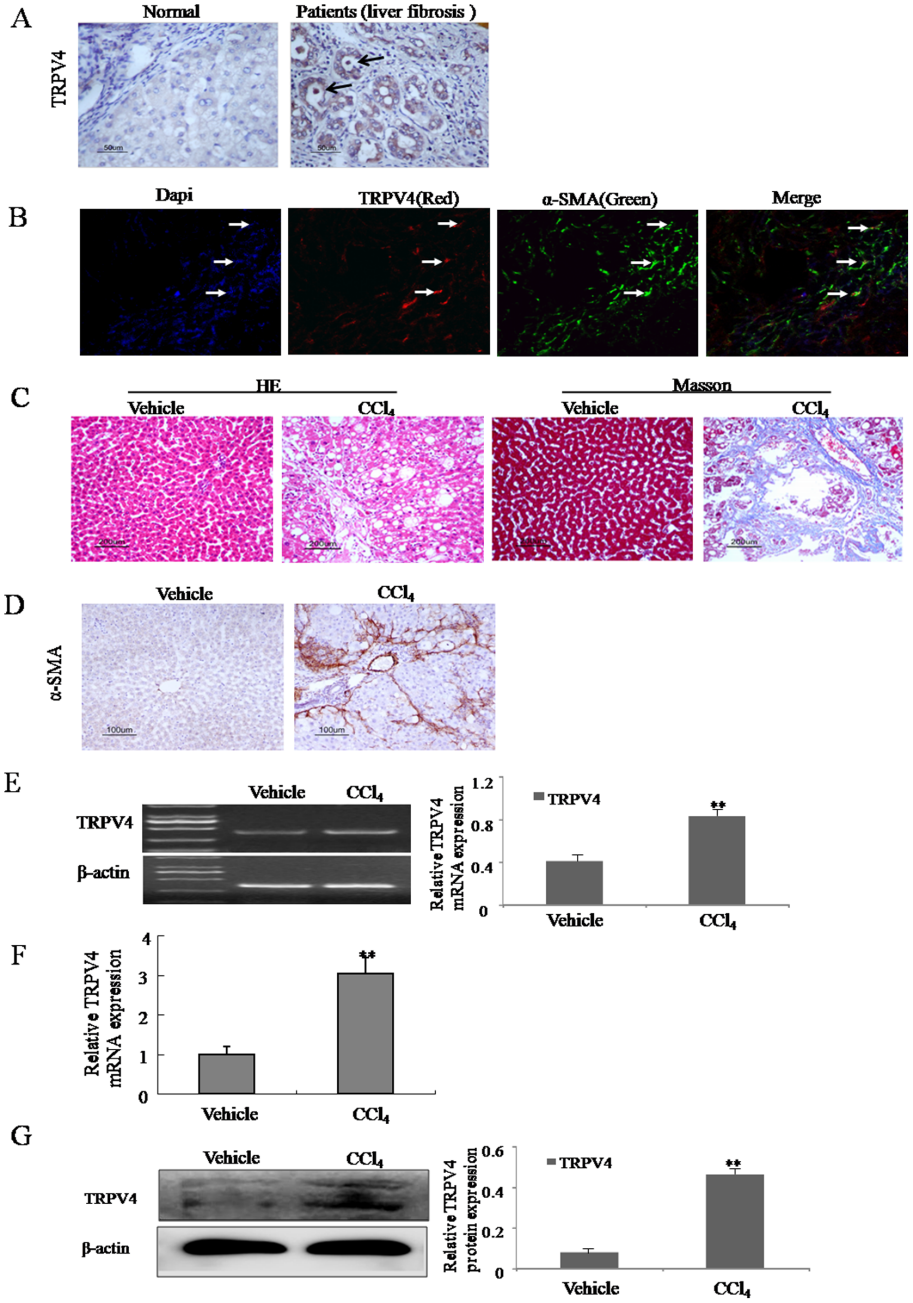
Upregulation of TRPV4 mRNA and protein in the liver tissues from liver fibrosis patients and CCl4-treated rats. A. The level of the TRPV4 was analyzed by immunohistochemistry in human normal liver and liver fibrosis patients. Representative views from each group are presented (original magnification, ×50). B. Liver tissues from liver fibrosis patients tissue were double stained with TRPV4 and α-SMA antibodies. Representative photomicrographs are shown (original magnification, ×40) in B. C. Pathology observation of the experimental rat liver sections stained with hematoxylin and eosin (H&E) staining and massion staining (×200). D. The level of the α-SMA was analyzed by immunohistochemistry in vehicle control livers and liver fibrotic tissue. Representative views from each group are presented (original magnification, ×50). E-F. Total RNAs were isolated from the livers of CCl_4_-treated rats or vehicle-treated groups. The expression of TRPV4 mRNA was assessed by RT-PCR(E) and realtime PCR(F). Representative images of three independent experiments are shown. *p<0.05, **p<0.01 vs. vehicle control. G. Whole-cell extracts were isolated from the liver tissues of CCl_4_-treated rats or vehicle-treated groups, and subjected to immunoblot for TRPV4 and β-actin control. Representative images of three independent experiments are shown. *p<0.05, **p<0.01 vs. vehicle control.

First, we investigated the expression of TRPV4 in the liver tissues from liver fibrosis patients. As shown in Fig. 1A, TRPV4 immunostaining signal was increased in the liver tissues from liver fibrosis patients compared to the normal liver. Additionally, the results of Immunofluorescence in human samples indicated that TRPV4(red) was highly expressed in α-SMA positive cells(green) in the fibrotic area, indicating HSC may be one of main cell types which express TRPV4 (Fig. 1B). Second, in order to identify the expression of TRPV4 in the liver tissues from CCl_4_-treated rats, the severity of liver fibrosis was determined by hematoxylin and eosin (H&E) staining and Masson's trichrome staining. H&E and Masson's trichrome staining showed normal lobular architecture with central veins and radiating hepatic cords in the vehicle group (Fig. 1C). The CCl_4_ treatment resulted in steatosis, inflammatory infiltration, and fibrosis in the main block of experiments of 12 weeks (Fig. 1C). Immunostaining from vehicle treated groups showed scarce α-SMA staining, whereas the liver tissues from CCl_4_-treated rats were extensively stained (Fig. 1D). Moreover, compared with the vehicle-treated groups, both RT-PCR and western blot analysis demonstrated that expression of TRPV4 mRNA and protein extracted from rat liver were elevated in CCl_4_-treated livers (Fig. 1E, F and G). Together, these data suggested that the TRPV4 was induced and may have certain functions in hepatic fibrosis.

### 3.2 Increasing expression of TRPV4 mRNA and protein during HSC activation

**Figure 2:**
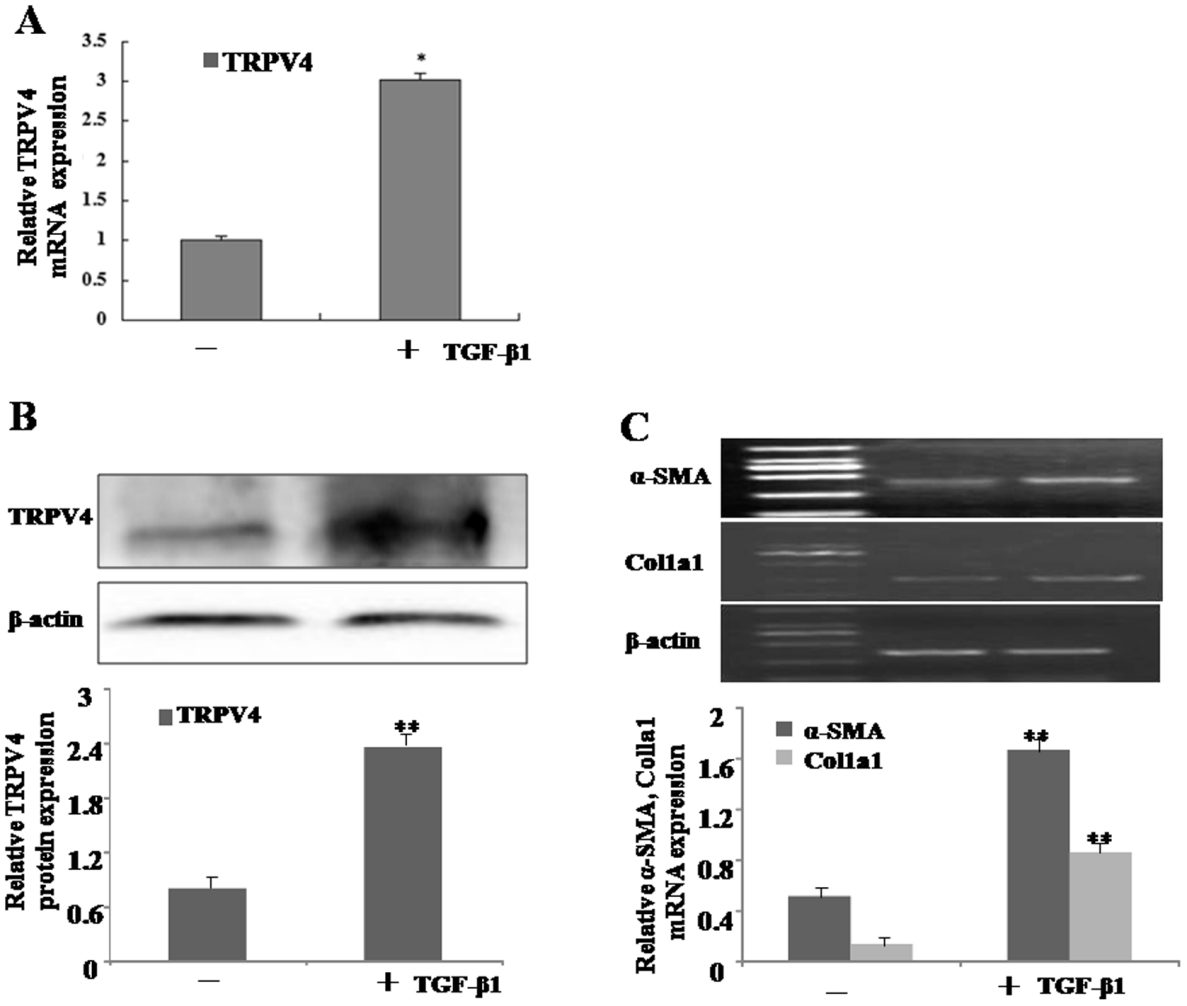
Increasing expression of TRPV4 mRNA and protein during HSC activation. A. Total RNAs were isolated from TGF-β1-treated HSC-T6 cells, and subjected to qRT-PCR analyses. Representative images of three independent experiments are shown. *p<0.05 vs. non-treated cells. B. Whole-cell extracts were isolated from TGF-β1-treated HSC-T6 cells, and subjected to Western blot analyses with TRPV4 and β-actin antibodies. Representative blots of three independent experiments are shown. **p<0.01 vs. non-treated cells. C. Total RNAs were isolated from TGF-β1 treated HSC-T6 cells at different time points. The expression of α-SMA and Col1a1 mRNA was assessed by RT-PCR. Representative images of three independent experiments are shown. **p<0.01 vs. non-treated cells.

In chronic liver injury, quiescent HSCs change into proliferative myofibroblast-like cells, which is a main source of extracellular matrix. To validate the alteration of TRPV4 gene expression during HSC activation also occurs in vitro, we examined the expressions of TRPV4 mRNA and protein in cultured rat HSC-T6 cells. As illustrated in Fig. 2A and B, HSC-T6 cells was incubated with 10 ng/ml TGF-β1 for 24 h, both mRNA and protein levels of TRPV4 were elevated. Additionally, α- SMA and Col1α1 expressions were increased according to the progression of HSC-T6 cell activation (Fig. 2C) and significantly correlated with the level of TRPV4.

### 3.3 Blockade of TRPV4 inhibited the proliferation and decreased α-SMA expression in activated HSC-T6 cells

**Figure 3:**
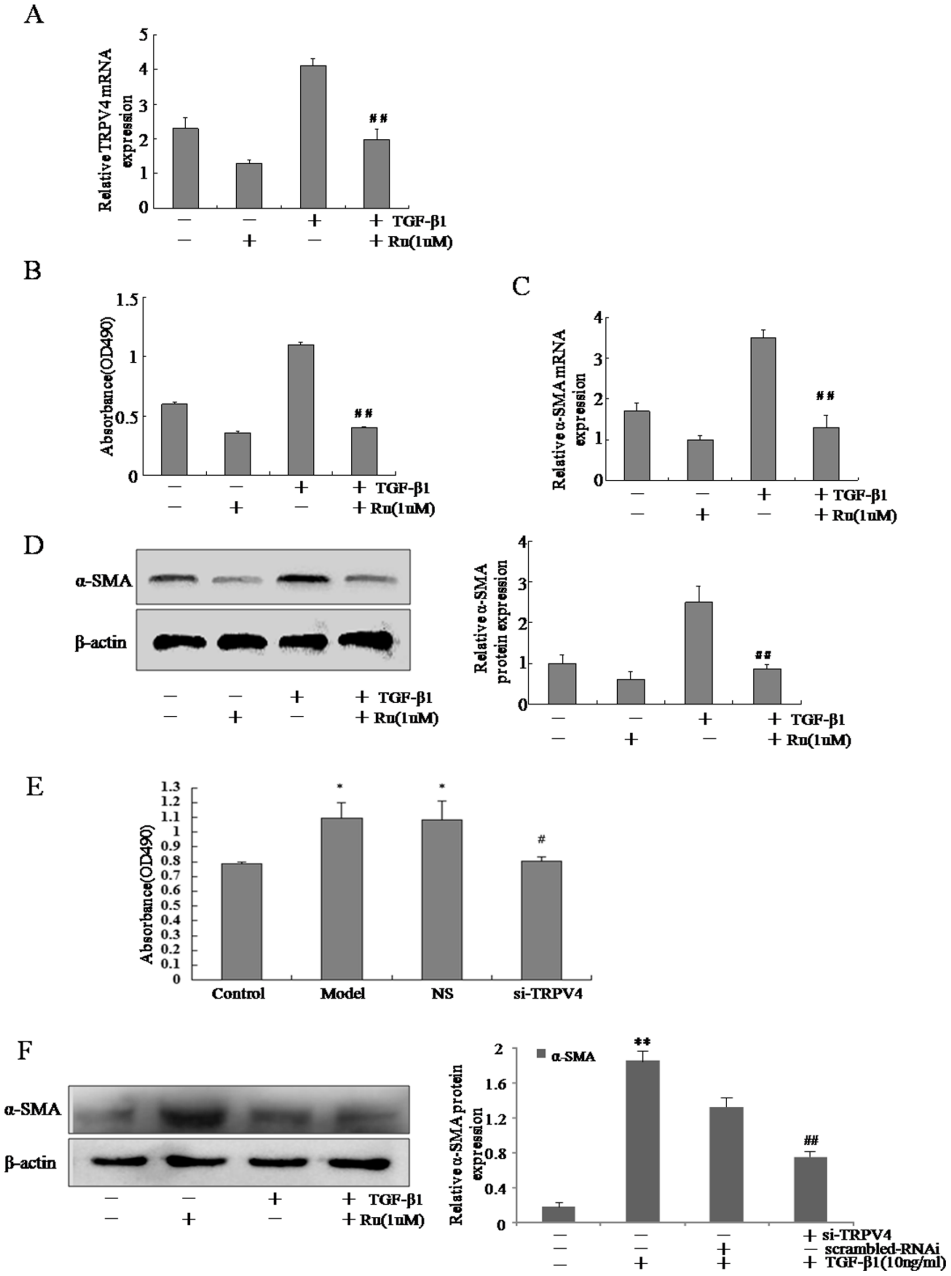
Blockade of TRPV4 inhibited the proliferation and decreased α-SMA expression in activated HSC-T6 cells. A. Total RNA extracts were made from HSC-T6 cells treated with or without TGF-β1 and Ru, and subjected to qRT-PCR analyses of TRPV4. Representative images of three independent experiments are shown. ^#^p<0.05 vs. TGF-β1-treated cells. B. HSC-T6 cells were seeded in triplicate on day 0 and incubated in DMEM containing 10% fetal bovine serum or same media supplemented with Ru for further 24 h. Proliferation was measured by adding 5 mg/ml MTT reagent per well and incubating it for 4 h. ^#^p<0.05 vs. TGF-β1-treated cells. C. Total RNA extracts were made from HSC-T6 cells treated with or without TGF-β1 and Ru, and subjected to qRT-PCR analyses of α-SMA. Representative images of three independent experiments are shown. ^#^p<0.05 vs. TGF-β1-treated cells. D. Whole-cell protein extracts were made from HSC-T6 cells treated with or without TGF-β1 and Ru, and subjected to Western blot analyses of TRPV4. Representative images of three independent experiments are shown. ^##^p<0.01 vs. TGF-β1-treated cells. E. HSC-T6 cells were treated with TGF-β1 for 48 h, followed by transfection with TRPV4-siRNA for an additional 48 h, and cell viability was determined by MTT assay. Mean±SE of two HSC preparations in quadruplets is shown; *p<0.05 vs. non-treated cells, ^#^p<0.05 vs. TGF-β1-treated cells. F. Whole cell extracts were isolated from TGF-β1-treated HSC-T6 cells with RNAi transfection, and subjected to Western blot analyses. Representative images of three independent experiments are shown. **p<0.01 vs. non-treated cells, ^##^p<0.01 vs. TGF-β1-treated cells.

To elucidate the functional role of TRPV4 during activation of HSC-T6 cells, Ruthenium Red (Ru), a common non-specific blocker of TRPV4 channel, was used to block this channel in TGF-β1-induced HSC-T6 cells. First, we tested the effect of Ru on the expression of TRPV4, as shown in Fig. 3A, Ru could inhibit the expression of TRPV4. Results of MTT assays demonstrated that Ru inhibited the proliferation of TGF-β1-treated HSC-T6 cells (Fig. 3B). To determine whether TRPV4 was capable of modulating α-SMA expression in vitro, we performed real time RT-PCR and Western blot assays on the liver tissues from CCl_4_-treated rats. The expression of α-SMA, an event associated with liver fibrosis, was suppressed in Ru-treated HSCs (Fig. 3C and D). To further establish the critical role of TRPV4 in the proliferation of activated HSC-T6 cells, siRNA specific for rat TRPV4 (TRPV4-siRNA) was used to knockdown the TRPV4 expression. As illustrated in Fig. 3E, MTT assay demonstrated that the viability of HSC-T6 cells was dramatically reduced after incubation with TGF-β1 and TRPV4-siRNA when compared to TGF-β1 and the scrambled-siRNA transfected cells. TGF-β1-activated HSC-T6 cells transfected with TRPV4-siRNA also expressed lower levels of α-SMA as compared to that transfected with the scrambled-siRNA (Fig. 3F). These data suggested that the blockade of TRPV4 channel inhibited TGF-β1-induced HSC-T6 cell proliferation and decreased the production of α-SMA in activated HSC.

### 3.4 Overexpression of miR-203 inhibits TGF-β1-induced HSC proliferation

**Figure 4:**
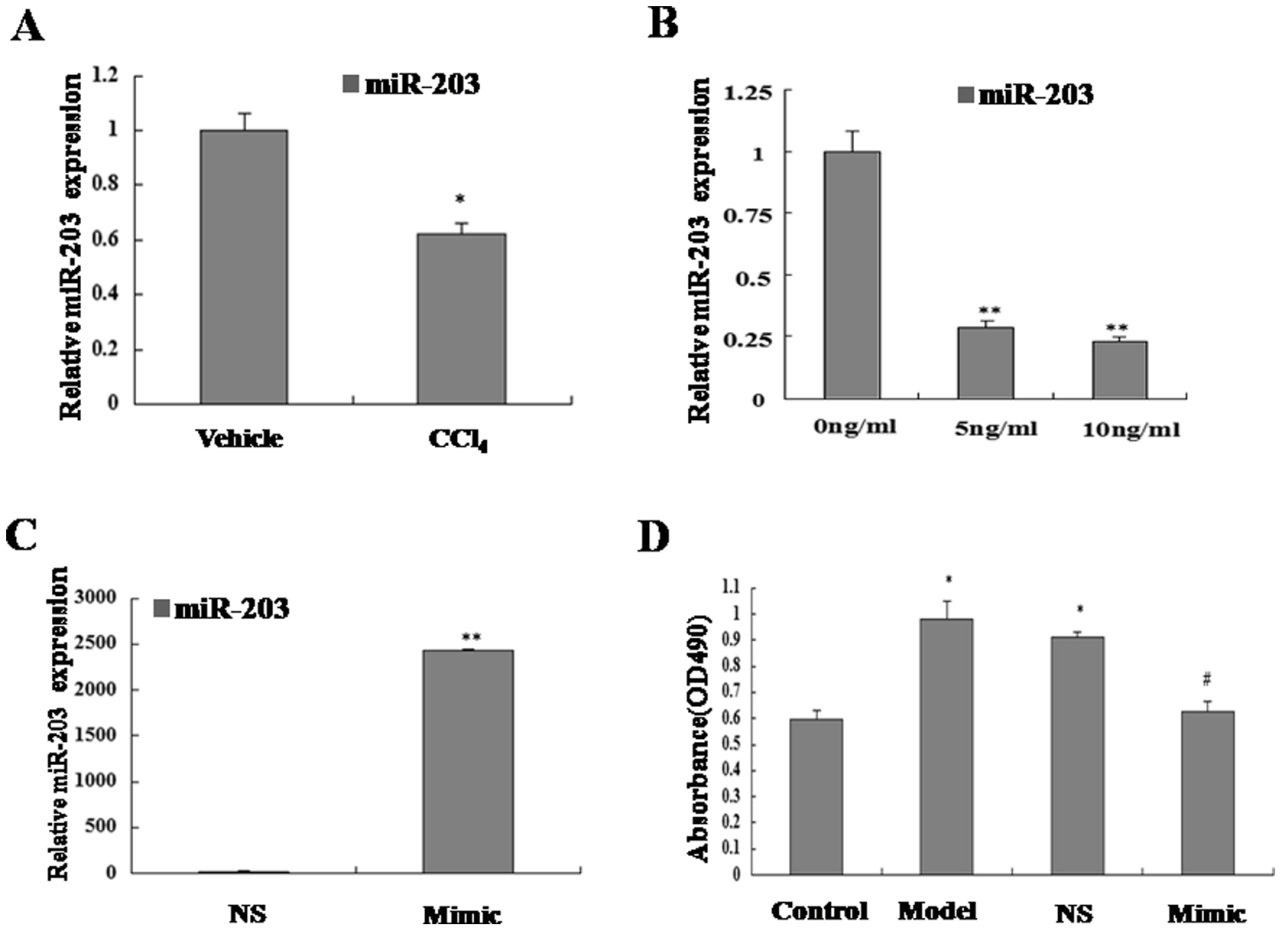
Overexpression of miR-203 inhibited of TGF-β1-induced HSC proliferation. A. Downregulation of miR-203 expression in liver fibrotic tissues compared with vehicle-treated groups. The miR-203 expression was analyzed by one-step quantitative real-time PCR. *P<0.05 vs vehicle control. B. Downregulation of miR-203expression in response to TGF-β1 (0, 5, 10 ng/ml) for 24 h. The miR-203 expression was analyzed by one-step quantitative real-time PCR. **P<0.01 vs non-treated cells. C. Upregulation of miR-203 expression in transfected HSC. The miR-203 expression of HSC was analyzed by one-step quantitative real-time PCR. **P<0.01 vs NS-miRNA. D. MiR-203 overexpression significantly inhibited proliferation of TGF-β1-induced HSC. The role of miR-203 in regulating TGF-β1-treated HSC proliferation was tested by MTT assay. The data represent the mean±SD of three different experiments. *P<0.05 vs control(non-treated cells), ^#^P<0.05 vs NS-miRNA.

miR-203, a tumor suppressor microRNA, is often silenced in different malignancies, but the roles of miR-203 during liver fibrosis remain obscure at the present. To our surprise, miR-203 was downregulated in rat liver fibrotic tissues compared with the normal liver tissues by one-step quantitative RT-PCR (Fig. 4A). To investigate whether miR-203 has a role in TGF-β1 induced HSC activation, we firstly sought to determine whether TGF-β1 regulates miR-203 expression in activated HSC. In this experiment, we observed a remarkable declined of miR-203 in response to TGF-β1, in addition, as shown in Fig. 4B, the expression of miR-203 was suppressed by TGF-β1 in a dose-dependent manner (range from 5 to 10 ng/ml at 24 h). These findings convincingly indicated that miR-203 was down-regulated dose-dependently in TGF-β1-induced HSC. In order to investigate the roles of miR-203 in regulating TGF-β1-induced HSC proliferation, we tested the effect of miR-203 overexpression on the proliferation of TGF-β1 induced HSC. The cells transfection with miR-203 mimics significantly increased mature miR-203 expression (Fig. 4C). The MTT assay showed that introduction of miR-203 caused a significant inhibition of cell proliferation in TGF-β1-induced HSC (Fig. 4D). These results indicated that overexpression of miR-203 inhibited TGF-β1-induced HSC proliferation.

### 3.5 TRPV4 is a Target of MiR-203 in HSC

**Figure 5:**
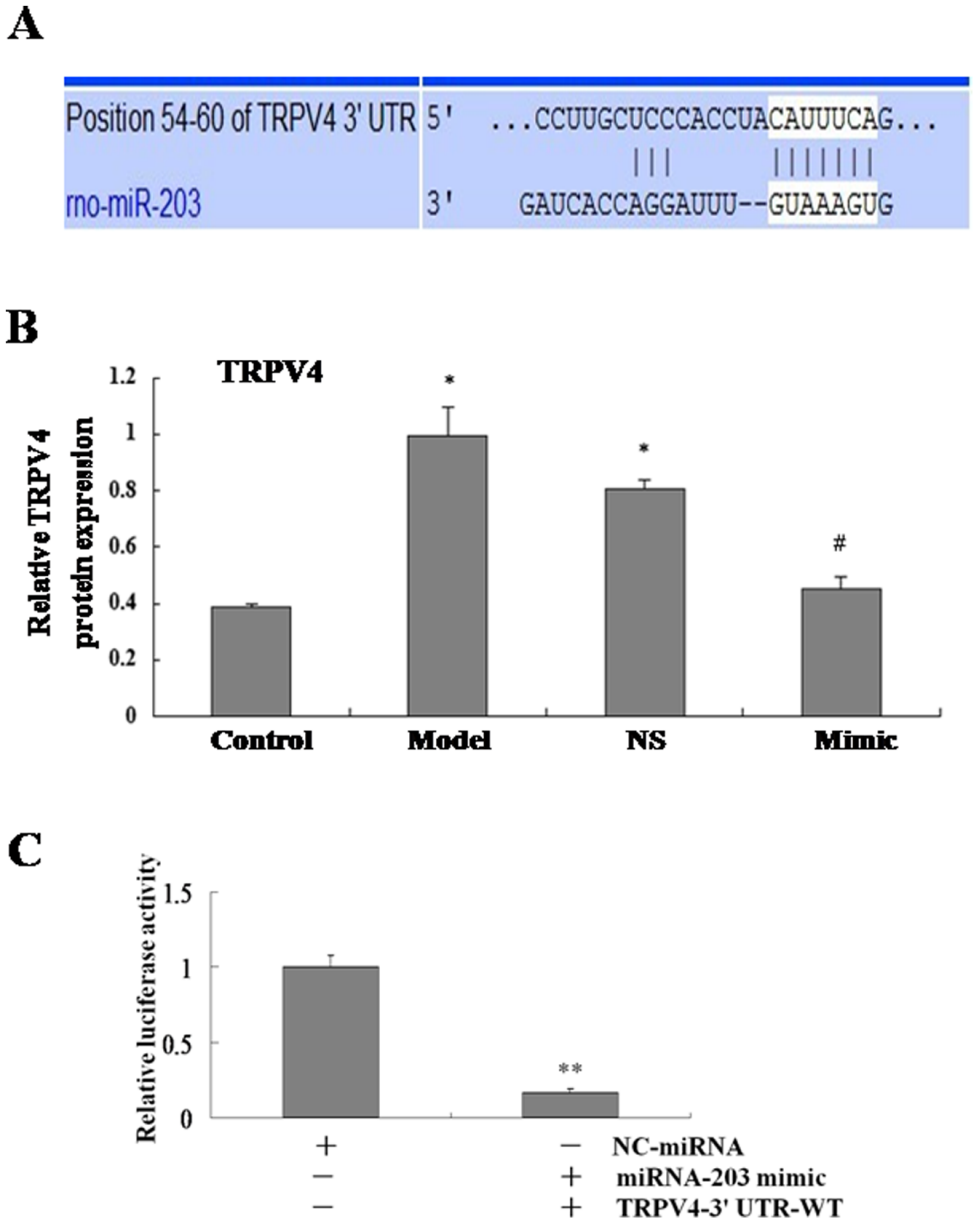
TRPV4 is a target of miR-203 in HSC. A. The region of the rat TRPV4 mRNA 3′UTR predicted to be targeted by miR-203. Three different bioinformatics approaches (miRbase, miRANDA, and Targetscan 5.1) were used for the target prediction. B. The TRPV4 mRNA expression was analyzed by real-time PCR. The results are expressed as relative expression against control expression. *P<0.05 vs control(non-treated cells), #P<0.05 vs model(TGF-β1-treated cells).C. Dual-luciferase reporter assays of TRPV4 in HSC-T6 cells. Reporter construct containing wt TRPV4 3′-UTR was cotransfected with miR-203 mimics or NC-miRNA. Relative luciferase activity was normalized to firefly luciferase. Data are representative of at least three separate experiments. *P<0.05, **P<0.01vs. NC-miRNA.

To determine the potential role of miR-203 in mediating TGF-β1-induced HSC proliferation, potential targets that are components of TGF-β1 signaling pathway were identified by using miRbase Targets, miRanda, and TargetScan 5.1. Among the candidate miR-203 targets, we paid more attention to TRPV4 (Fig. 5A). MiR-203 mimics was transfected into TGF-β1-treated HSC. Real-time PCR analysis revealed that TRPV4 expression was significantly lower in miR-203 transfected HSC compared with the NS-miRNA-transfected cells (Fig. 5B). To determine whether TRPV4 gene is a true directly target of miR-203, luciferase reporter vectors encoding a fragment of wild TRPV4 mRNA 3′-UTR, namely, TRPV4-wt was subcloned into a reporter vector downstream of the luciferase gene in HSC. Luciferase reporter assays showed that the relative luciferase activity of the reporter that contained wild-type 3′-UTR of TRPV4 mRNA was significantly decreased in miR-203-overexpressed cells compared to control cells (Fig. 5C). These results demonstrated that TRPV4 is likely a direct target of miR-203.

## Discussion and Conclusion

Activation of HSC to a myofibroblast-like phenotype is the pivotal event in liver fibrogenesis [12], [13]. HSC activation is characterized by enhanced cell proliferation and over-production of ECM. Recent studies indicated that ECM stiffening influenced the fibroblast differentiation to myofibroblasts, resulting in further increases in ECM deposition leading to the progression of the disease [14], [15]. Therefore, inhibition of HSC activation/proliferation and blockage of ECM production are important strategies for therapeutic intervention. However, the molecular mechanisms for regulation of HSC activation/proliferation have not been well elucidated. Here, our study provided the initial evidence that, as the target of miR-203, TRPV4 is significantly increased in fibrotic rat livers and plays a critical role in controlling HSC activation.

TRPV4 cation channel, a member of the TRP vanilloid subfamily, is widely expressed in a broad range of tissues [16], [17]. Subsequent studies have shown that TRPV4 channels possess multiple activation and regulatory sites that allow them to integrate distinct physical and chemical stimuli from the environment, offering a wide range of possible physiological roles, such as cell proliferation, survival, differentiation, migration, adhesion [18], [19], [20], [21]. It is reported that TRPV4 regulates cardiac fibroblast differentiation to myofibroblasts by integrating signals from TGF-β1 [22], [23]. And an exciting possibility raised by Arniges opens up in which TRPV4 could become a translational target in cystic fibrosis [24]. In the present study, we found that TRPV4 channel was dramatically increased in the liver tissues from patients with liver fibrosis and CCl_4_-treated rats in vivo and its expression was strongly correlated the HSC activation identified by the induction of α-SMA and Col1α1. And we found that TRPV4 is increased in HSC-T6 cells is response to TGF-β1 stimulation. These results suggested that TRPV4, in activated HSCs and in fibrotic livers, was likely upregulated. TRPV4 was associated with cell proliferation in various cell types. Accordingly, blockade of TRPV4 channels leads to the inhibition of proliferation in different cell types. In the present study, exposure of activated HSC-T6 cells to Ruthenium Red (Ru), dramatically decreased the cell viability. Additionally, knockdown of TRPV4 with siRNA strongly inhibited the proliferation of activated HSC-T6 cells. Moreover, induction of collagen and α-SMA is considered as a marker of HSC activation and liver fibrosis. Expression level of Col1α1 and α-SMA were upregulated in activated HSC-T6 cells and CCl_4_-treated liver. In current study, results indicated that the blockade of TRPV4 with Ru or siRNA down-regulated the expressions of Col1α1 and α-SMA in activated HSC-T6 cells. In combination with MTT results, we suggested that TRPV4 may be required for HSC activation and proliferation.

microRNAs are a group of small non-coding RNA, which can regulate expression of hundreds of target genes and thereby influencing various biologic processes such as cellular proliferation and differentiation [25]. Accumulating studies have demonstrated that miRNAs played important roles in regulating HSC functions such as cell proliferation, differentiation, and apoptosis [26], [27], [28]. For an instance, miR-146a inhibits TGF-β1-induced HSC differentiation, at least in part, via decreasing the expression of SMAD4 [27]. Recently the microarry analysis revealed that the expression of miR-203 was significantly downregulated in chronic liver disease and HCC [29], [30]. It has also been shown that miR-203 could up-regulate NO expression in female rat MCCs via targeting TRPV4 [31]. The present study was destined to determine whether miR-203 could play a functional role in regulating HSC proliferation in response to TGF-β1 stimulation. In our study, miR-203 expression was downregulated dose-dependently in response to TGF-β1, MTT assay showed that transfection of miR-203 mimics led to a significant inhibition of cell proliferation in TGF-β1-treated HSC. Moreover, our experiments demonstrated that miR-203 overexpression resulted in significantly decreased TRPV4 expression, suggesting the inhibitory role of miR-203 in liver fibrosis may be correlated to function of TRPV4.

**Figure 6:**
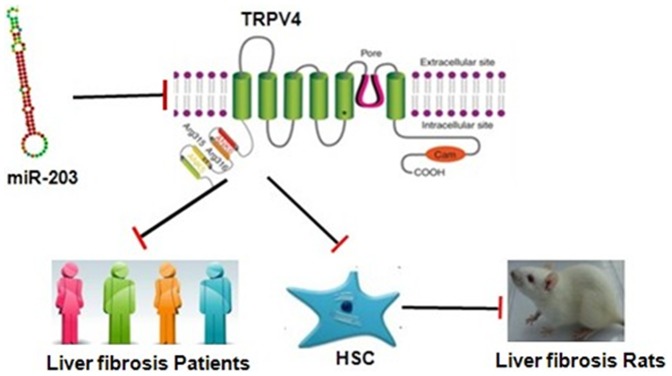
Overview of TRPV4 on liver fibrosis.

In summary, our findings in the present study suggested that TRPV4 may play a pivotal role during hepatic stellate cell activation. The blockade of TRPV4 channels offset TGF-β1-induced HSC-T6 cell proliferation, indicating the potential of TRPV4 as a therapeutic target for liver fibrosis. Moreover, it appeared that the expression of TRPV4 was directly regulated by miR-203 in TGF-β1-induced HSC (As shown in Fig. 6). To our knowledge, this is the first report focusing on the role of TRPV4 in liver fibrosis. This study gave us novel insights into the potential roles of TRPV4 channels in HSC activation, further functional analysis to determine the precise role of TRPV4 in HSC bioactivity could provide a novel therapeutic approach for treating hepatic fibrosis.
